# The CirPAD, a circular 1.4 M hybrid pixel detector dedicated to X-ray diffraction measurements at Synchrotron SOLEIL

**DOI:** 10.1107/S1600577521012492

**Published:** 2022-01-01

**Authors:** Kewin Desjardins, Cristian Mocuta, Arkadiusz Dawiec, Solenn Réguer, Philippe Joly, Jean-Michel Dubuisson, Filipe Alves, Arafat Noureddine, Frédéric Bompard, Dominique Thiaudière

**Affiliations:** a Synchrotron SOLEIL, Saint-Aubin, 91192 Gif-sur-Yvette, France; b Cegitek, La Plaine du Caire, Le Clos du Rocher, Bat 9, 13830 Roquefort-la-Bédoule, France

**Keywords:** hybrid pixel X-ray detector, 2D X-ray diffraction imaging, X-ray powder diffraction, XPAD, CirPAD, DiffAbs

## Abstract

One of the challenges of all synchrotron facilities is to offer the highest performance detectors for all their specific experiments, in particular for X-ray diffraction imaging and its high throughput data collection. In that context, the DiffAbs beamline, the Detectors and the Design and Engineering groups at Synchrotron SOLEIL, in collaboration with ImXPAD and Cegitek com­panies, have developed an original and unique detector with a circular shape. This detector is based on the hybrid pixel photon-counting technology and consists of the specific assembly of 20 hybrid pixel array detector (XPAD) modules. This article aims to demonstrate the main characteristics of the CirPAD (for Circular Pixel Array Detector) and its performance – *i.e.* excellent pixel quality, flat-field correction, high-count-rate performance, *etc*. Additionally, the powder X-ray diffraction pattern of an LaB_6_ reference sample is presented and refined. The obtained results demonstrate the high quality of the data recorded from the CirPAD, which allows the proposal of its use to all scientific communities inter­ested in performing experiments at the DiffAbs beamline.

## Abstract 2

The DiffAbs beamline, the Detectors and the Design and Engineering groups at Synchrotron SOLEIL, in collaboration with ImXPAD and Cegitek com­panies, have developed an original and unique detector with a circular shape. This detector is based on the hybrid pixel photon-counting technology and consists of the specific assembly of 20 hybrid pixel array detector (XPAD) modules.

## 1. Introduction

The French synchrotron facility, Synchrotron SOLEIL, offers a large panel of synchrotron experiments to the scientific community thanks to its 29 different beamlines. Among these, the DiffAbs beamline (Baudelet *et al.*, 2005 ▸; Coati *et al.*, 2017 ▸) provides a monochromatic X-ray beam (tunable in the 3–23 keV energy range) from a bending magnet source. DiffAbs can operate in two modes: (i) the ‘standard beam’ mode, obtained using the main optics [a double Si(111) crystal monochromator plus two long mirrors consisting of 50 nm Rh-coated Si single crystals], and (ii) the ‘microbeam’ mode obtained by adding secondary focusing optics (two mirrors in Kirkpatrick–Baez geometry or Fresnel-zone plates). Regardless of the X-ray beam energy, at the sample position (corresponding all the time to the centre of a six-circle diffractometer), the resulting beam size is about 250 µm × 250 µm (Full Width at Half-Maximum, FWHM) and about 5 µm × 5 µm, respectively, for the mentioned functioning modes. The typical divergence of the X-ray beam amounts to 0.01° × 0.1° (vertical × horizontal) and is increased up to about 0.2° × 0.2° for the ‘standard beam’ and ‘microbeam’ modes, respectively. At the critical energy of the bending magnet source (8.6 keV), the measured photon flux at the sample position is 4 × 10^12^ photons s^−1^ in the ‘standard beam’ configuration. One of the originalities of DiffAbs is its ability to study a large variety of materials, such as metallic alloys, semiconductors/oxides, polymers (natural, artificial and syn­thetic), biominerals, glasses, ceramics, *etc*., with various homo­geneous or heterogeneous forms, such as polycrystalline, epitaxial or amorphous. Most of the time, these materials are placed inside a specific environment, such as furnaces, mech­ani­cal loading devices, chemical or electrochemical cells, *etc*. In other words, DiffAbs allows the combination of a set of X-ray-based analytical techniques, such as X-ray diffraction (XRD), wide-angle X-ray scattering (WAXS) and resonant X-ray diffraction like anomalous X-ray diffraction (AXRD) or diffraction anomalous fine structure (DAFS), but also X-ray absorption (XAS) and X-ray fluorescence (XRF) spectroscopies.

To obtain information about the structural evolution or phase transformations of polycrystalline (textured or not) and/or amorphous materials using XRD or, more generally, WAXS, the detection system must combine several crucial characteristics: fast readout with high sensitivity, large dynamic range, low noise and a large active area. Nowadays, to address these particularly on the DiffAbs beamline, only 2D detectors using Hybrid Pixel photon-counting technology (Brönnimann & Trüb, 2015 ▸) are considered and used routinely (Mocuta *et al.*, 2018 ▸). Compared to the microstrip detector (Bergamaschi *et al.*, 2010 ▸), which remains faster and largely used for 1D powder X-ray diffraction (PXRD), the pixelated technology allows detectors with a large sensitive area to be made, providing information in both directions. Furthermore, with a small pixel size (down to 55 µm), their count-rate capability is higher (more than 1 × 10^9^ photons s^−1^ mm^−1^). Moving the detector around the sample to collect the signal over a wide-angle domain is also an inter­esting option, although to fit the experimental needs, similar to the strip detector, 2D Hybrid Pixel detectors can be assembled into a curved shape. As an example, an arrangement of 120 DECTRIS PILATUS 2100K detectors (modules with a 172 µm pixel size) in a semicylindrical shape (Wagner *et al.*, 2016 ▸) of 250 mm radius has been implemented for in-vacuum macromolecular crystallography at Diamond Light Source (DLS). The result is a detection geometry with a wide collection angle of scattered signal of ±100°. A smaller-radius combination of 24 modules of a 3Medipix3RXv2 detector (Gimenez *et al.*, 2019 ▸), covering an angle of 100°, is also used at the DLS in order to investigate samples *via* X-ray Pair Distribution Function measurements. This kind of curved design clearly allows the ranges of scattering angles used for the collection of data to be increased dramatically without scanning of the position of the detector. This geometry could also be optimized to limit shading and dead zones using the tiling method or edgeless sensor technology, allowing it to be tightly packed together from all sides, as done on the WidePix curved detector (Jakubek *et al.*, 2014 ▸).

However, up to now, there was no detector available at the beamline which could combine high-speed acquisition (higher than 500 Hz to follow transformations in the studied materials), short acquisition time (to perform time-resolved acquisition in stroboscopic experiments) and very large area (more than 100° to record full structural information with an angular resolution in the order of 0.01°) with increased flexibility of use (with two degrees of freedom). For this reason, the Detectors group and DiffAbs beamline on one side, and the Cegitek com­pany (formerly imXPAD) on the other, developed an original high-speed 2D detector based on a silicon sensor hybrided into an XPAD3.2 readout chip (Pangaud *et al.*, 2007 ▸), with a particular geometry covering a very wide angular range.

This 2D detector, named CirPAD, for Circular hybrid Pixel Array Detector, is basically an assembly of 20 XPAD-S70 modules (Medjoubi *et al.*, 2010 ▸) tiling arranged along their long sides/axes on a circular arch geometry. The result is a high-speed movable detector with 1.4 MP covering a wide collection angle of 135° with a radius of 645 mm and a pixel angular opening of 0.0115°.

This article aims at presenting the general characteristics of the CirPAD. Firstly, the main specifications of the XPAD readout chips will be summarized. Detailed information about their assembly is reported, including the electronics and the mechanical design, with particular attention paid to the overlaps between modules (to minimize the ‘dead zones’) and the different holders and frame (from the module holder to the installation of the CirPAD on the six-circle diffractometer of the beamline). A third part deals with the performance of the detector and, finally, a first reconstructed X-ray diffraction pattern from a certified LaB_6_ powder from the National Institute of Standards and Technology (NIST) will be shown (Black *et al.*, 2020 ▸).

## 2. The CirPAD hardware

### 2.1. General architecture and characteristic

The XPAD hybrid pixel detectors with different sizes and geometries are used routinely on several beamlines at SOLEIL for different applications that take advantage of the unique features of the XPAD readout chip and, on some occasions, of a very specific development of the detector firmware. Examples of the use of the detector include time-resolved diffraction experiments on the CRISTAL beamline, which requires an extremely short gating time, in the range of a few tens of nanoseconds (Laulhé *et al.*, 2012 ▸; Fertey *et al.*, 2013 ▸), surface diffraction with a real-time beam attenuation control on the SIXS beamline (Dawiec *et al.*, 2016 ▸), XRD maps of heterogeneous materials, such as ancient materials and medical samples, on the PUMA and DiffAbs beamlines (Guériau *et al.*, 2020 ▸; Kergourlay *et al.*, 2018 ▸), and, of course, the already mentioned hard-X-ray diffraction on the DiffAbs beamline (Mocuta *et al.*, 2018 ▸).

**Figure 1:**
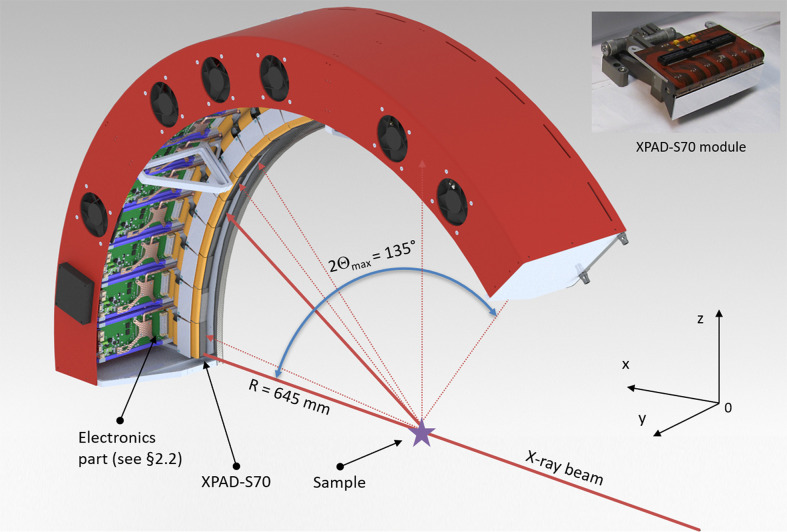
Schematics of the CirPAD, showing (top-right inset) a photograph of an XPAD-S70 module assembly with a silicon sensor.

The CirPAD is a new member of the XPAD detectors family that is the largest XPAD-based detector to date. It is com­posed of an assembly of 20 modules, so-called XPAD-S70 (67200 pixels per module), with a specific architecture and mechanical circular design, as illustrated in Fig. 1 ▸. Finally, the CirPAD consists of over 1.344 million pixels arranged in a matrix of 120 × 11200 pixels.

**Figure 2:**
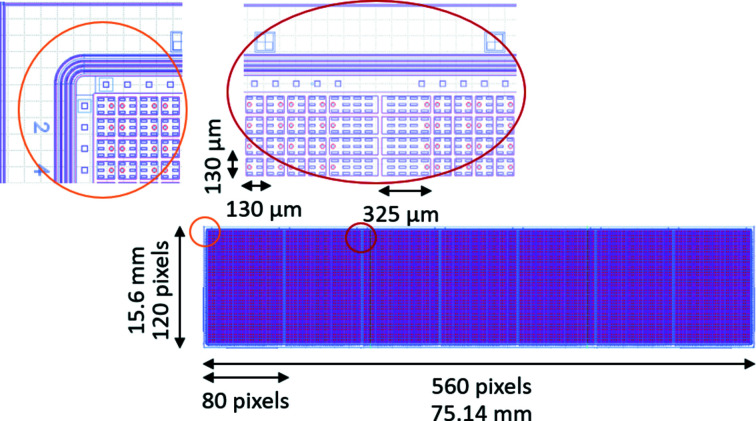
Schematic representation of the XPAD-S70 module. Magnifications of the guard ring and double pixel part are shown (orange and red colours, respectively).

The XPAD-S70 (see top-right inset in Fig. 1 ▸) is a hybrid detector module com­posed of a 500 µm thick single silicon sensor bump bonded to seven XPAD3.2 single photon counting readout chips (Fig. 2 ▸). The single XPAD3.2 readout chip consists of square pixels with a 130 µm pitch arranged in 120 lines of 80 pixels, which corresponds to 15.6 mm × 10.4 mm. The sensor pixels that cover the inter­chip gap (the space between two adjacent readout chips) are 2.5 times larger in the horizontal direction, hereafter ‘double’ pixels (see magnified areas in Fig. 2 ▸). The active area of the sensor is surrounded by the 550 µm region that accommodates several guard rings connected to the ground. The double pixels and the guard ring give rise to a larger value of counted photons in these regions, which can be corrected post-acquisition by inserting virtual columns of pixels and flat-field correction. The resulting effective module size is 16.8 mm × 76.24 mm, while the active area is 15.6 mm × 75.14 mm (91.6% ratio). The active area of a single module corresponds to a matrix of 578 × 120 square pixels (including an inter­chip gap correction, *i.e.* three pixels between two adjacent chips), therefore a total of 69360 pixels.

The analogue front-end of every pixel is com­posed of a low-noise Charge-Sensitive Amplifier (CSA) followed by an Operational Transconductance Amplifier (OTA) and a cur­rent mode discriminator with a programmable threshold. The energy threshold value is set globally for all the pixels by a single 8-bit global DAC (Digital to Analogue Converter) and is adjusted locally in every pixel with a 6-bit Local DAC (DACL). The discriminator output feeds a 12-bit depth counter, with an extra 13th bit called the overflow (OVF) that changes its value each time 4096 photons are accumulated in a pixel. The detailed characteristics of the XPAD3.2 readout chip and the XPAD-S70 module have already been discussed extensively elsewhere (Pangaud *et al.*, 2007 ▸; Medjoubi *et al.*, 2010 ▸), and are summarized in Table 1 ▸. The particularity of the XPAD3.2 readout chip resides in the fact that the overflow bit is accessible independently of the rest of the counter during image acquisition; this allows the dynamics of the measured signal to be extended to a wider range (up to 32 bits), limited only by the processing electronics. This is achieved by periodic readout of the OVF bit from all pixels at a rate that is higher than the pixel maximum counter-filling rate, *i.e.* every 4 ms. The OVF readout requires 200 µs during which the counters are disabled for photon counting and therefore introducing 5% of the dead time.

In order to obtain a uniform response from all pixels and therefore an optimal quality of images, the intrinsic pixel-to-pixel dispersion due to the mismatch in offsets and gain must be corrected. The correction process is called the threshold calibration. The XPAD detector offers two modes:

• The Over-The-Noise (OTN) calibration – the correction of the offsets, for which the threshold of every pixel is set just above the electronic noise. The OTN calibration does not require the presence of the X-ray beam.

• The beam calibration – the correction of both the offsets and the pixel gain dispersions. The beam calibration is done by illuminating all pixels with monochromatic X-rays and the threshold for every pixel is set according to the incident beam energy. The beam calibration is always performed at half of the working energy to minimize the charge-sharing effect between adjacent pixels (Mathieson *et al.*, 2002 ▸).

Additionally, the calibration can be done using several predefined settings of the analogue front-end that are a com­promise between the maximum photon count rate and noise, *i.e.* SLOW and FAST settings. The SLOW setting is optimized to work with lower energy photons (down to ∼4 keV) but at the cost of the maximum count rate (8.4 × 10^5^ photons s^−1^ pixel^−1^; see §3.4), while the FAST setting allows a maximum speed of the detector (2.5 × 10^6^ photons s^−1^ pixel^−1^; see §3.4), but with increased electronic noise and therefore higher minimum threshold settings (down to ∼5 keV).

Also, the CirPAD, like all XPAD detectors, can be controlled with either proprietary Cegitek software or with a TANGO LIMA controls device (https://lima1.readthedocs.io/en/latest/) that connects directly to the Cegitek server *via* the corresponding Application Programming Inter­face (API). Both inter­faces provide full access to the detector and allow the detector to be operated in four different acquisition modes:

• Standard Mode: the detector counts all the incoming photons during user-defined time. The image depths in the Standard Mode can be 12 bits (no OVF readout) or 16/32 bits with maximum frame rates of 500 and 250 Hz, respectively. The Standard Mode acquisition can be triggered with software or hardware signal.

• Burst Mode: like the Standard Mode, but instead of transferring the acquired images (up to 980) to the server, they are stored locally which significantly increases the frame rate up to 700 Hz.

• Stacking Mode: allows up to 980 images in total to be accumulated (in 16 bits, frame rate up to 700 Hz) and dis­tributed, by order of acquisition, into stacks of user-defined size. This accumulation is performed by the processing electronics of the detectors module (Fertey *et al.*, 2013 ▸).

• Single Bunch Mode: acquisition with an ultra-short gate, down to 100 ns for time-resolved applications (Fertey *et al.*, 2013 ▸; Ors *et al.*, 2019 ▸).

### 2.2. CirPAD assembly and geometry

Each XPAD-S70 module is glued to a dedicated graphite holder (see top-right inset in Fig. 1 ▸) that provides good mechanical rigidity and heat dissipation, while maintaining a light weight com­pared to alternative solutions, *e.g.* copper or aluminium supports. The electrical connection between the module and the electronic board is ensured by custom Kapton flexible cables that keep the acquisition board at the optimal angle and relaxes mechanical constraints.

**Figure 3:**
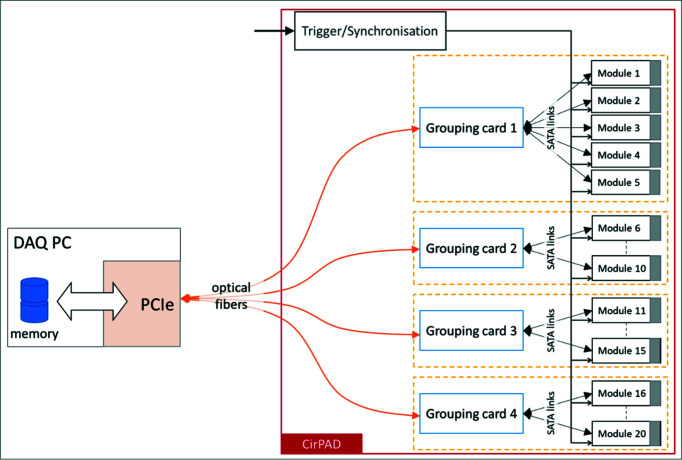
Readout architecture of the CirPAD.

Each XPAD-S70 module is connected one-by-one to a dedicated acquisition and processing board build around an Altera CycloneIV GX FPGA (Field-Programmable Gate Array). These boards are grouped in fives and are connected to a dedicated regrouping board *via* copper Serial Advanced Technology Attachment (SATA) links. The com­plete detector assembly consists of four groups of five and thus a total of 20 XPAD-S70 modules that are operated independently and simultaneously (Fig. 3 ▸). The CirPAD is directly connected, *via* optical fibers, to a Linux PC server equipped with a dedicated Peripheral Component Inter­connect Express (PCIe) board. This allows the server to be installed outside the experimental hutch to limit heating and radiation damage.

In addition to the com­plexity related to the readout of 20 modules, the most important challenge of the CirPAD manufacture is based on its unique geometry, which required the design and realization of a precise mechanical frame which must ensure several critical features, such as: (i) accommodate 20 XPAD-S70 modules, the acquisition boards of the 20 modules, four regrouping boards, power supply, synchronization board and cooling system; (ii) high mechanical stability of all 20 modules; (iii) high precision of the positioning of each of the XPAD-S70 modules, with individual adjustment to minimize the space between them; (iv) heat dissipation to maintain the stable temperature of the detector modules (*via* water cooling) and all electronic com­ponents (*via* air cooling).

**Figure 4:**
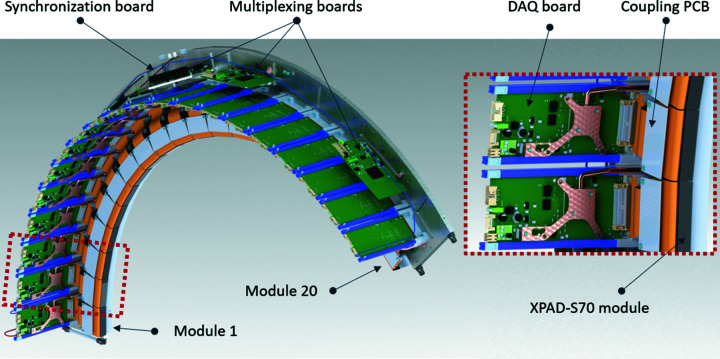
CirPAD frame and mounting of the XPAD-S70 modules. The 20 XPAD-S70 modules associated with the 20 FPGA acquisition board (DAQ) are also shown. The multiplexing boards are placed behind. The power supply and cables are not shown. The right-side inset shows a magnification of two consecutive modules.

The frame was made of aluminum (AlMg4 5086), which has good thermal and mechanical properties, allowing at the same time the final weight to be limited. The main inter­nal electronic com­ponents are visible in Fig. 4 ▸.

**Figure 5:**
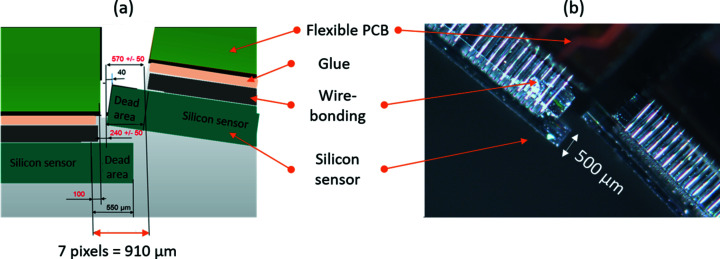
The overlap of two XPAD-S70 modules, showing (*a*) a schematic view and (*b*) a photograph of two consecutive modules.

As mentioned earlier, the frame provides the mechanical support for 20 modules, which are placed side-by-side over a circle with a radius of 645 mm and covering an angular range of about 135°. To minimize the dead zones, the adjacent modules overlap by a region with a maximum of 910 µm (equivalent to 7 pixels). The circular geometry forces each module to be tilted at an angle of 6.7° with respect to the previous one and to be aligned using two dedicated flexure-based mechanisms made of titanium (TA6V). The first one allows the rotation angle to be adjusted within a 1.5° angular range and with a 0.01° accuracy, while the second one allows lateral adjustment within a 600 µm range (±300 µm) with 5 µm accuracy. A schematic representation of the positions of two adjacent modules with the sensor overlap and a photograph are shown in Fig. 5 ▸.

The CirPAD is equipped with two independent cooling systems. Air cooling dissipates the heat from all the electronic com­ponents, mainly from all the FPGA boards, the power board and the CirPAD modules. An additional water-cooling circuit integrated within the frame is also available to further decrease and stabilize the temperature of the XPAD-S70 modules.

All graphite module holders are directly related to the frame with a copper braiding. Additionally, the total thermal expansion of each module has been estimated using *ANSYS* simulation software (https://www.ansys.com) and does not exceed a few microns with respect to the width and thickness of the module, and a few dozen microns with respect to the longitudinal direction.

### 2.3. CirPAD DiffAbs beamline experimental station

**Figure 6:**
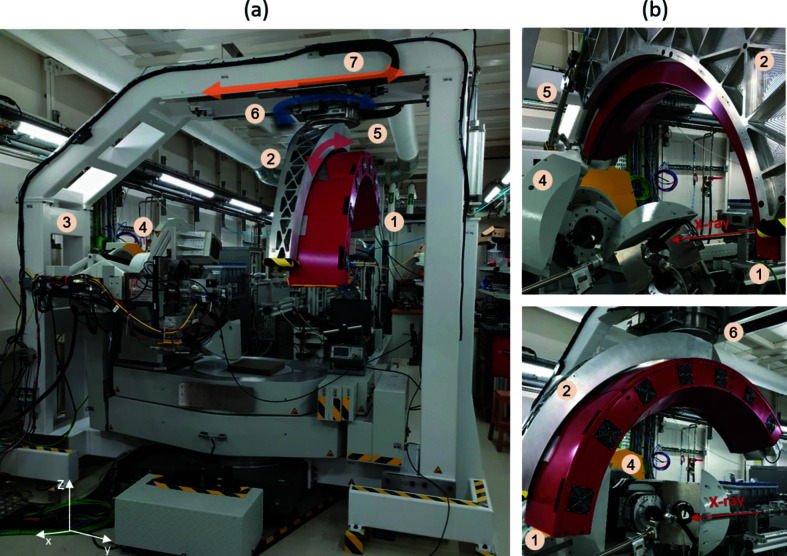
(*a*) The CirPAD installed on the diffractometer on the DiffAbs beamline at SOLEIL, showing (**1**) CirPAD, (**2**) arch, (**3**) crane, (**4**) diffractometer, (**5**) motorized rotation (16.5° amplitude, around the *y* axis), (**6**) motorized rotation (120° amplitude, around the *z* axis) and (**7**) manual translation. (*b*) The CirPAD in the two different working positions, with an angle of 90 or 0° between its detection plane and the direction of the incident beam.

The CirPAD frame with all detector com­ponents assembled has an overall volume of 0.85 m^3^ and a total weight of 70 kg. It has been mounted on a specially designed arch-shaped mechanical and handling frame. Particular attention was paid to maintain the position and angle of the detector according to the potential deformations due to its weight and possible thermal dilatation. Similar to the work performed on the CirPAD frame, several *ANSYS* simulations have been carried out to ensure that the mechanical deformation did not exceed the pixel size. Finally, the CirPAD is installed on the detector crane which surrounds the six-circle diffractometer that is already available at the DiffAbs beamline. A photograph of the CirPAD mounted on the crane is shown in Fig. 6 ▸. Two motorized rotation stages have been added to operate in a large angular range, up to 16.5° around the *y* axis and from −40 to +95° around the *z* axis. Furthermore, from a manual translation, the CirPAD can be removed from the measurement position in order to perform experiments requiring the use of other detectors. Table 2 ▸ summarizes the main characteristics of the CirPAD and its experimental capabilities.

## 3. CirPAD characterization

### 3.1. Selection of modules and initial acceptance tests

**Figure 7:**
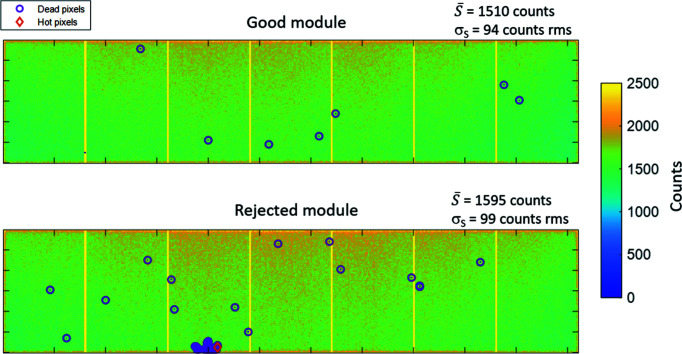
An example of good (upper image) and rejected (lower image) modules after first qualification tests with 14.1 keV X-ray photon energy.

Characterization, performance and acceptance tests have been carried out at the Detectors group laboratory to verify the quality and confirm the specifications (see Table 1 ▸) of each individual XPAD-S70 module prior to installation inside the com­plete CirPAD package. The first step of module selection was based on a visual inspection of each module in order to identify any potential physical damage. In a second step, the response to the X-ray beam has been measured for over 25 modules that have been manufactured for this detector, which allowed the selection of those with the best characteristics. After performing the OTN calibration, every module has been exposed to a broad size and monochromatic X-ray beam of 14.1 keV energy from the fluorescence target (strontium *K*α line) which has been placed in the direct beam path of a laboratory X-ray generator. One of the main qualification factors of the investigated modules was the number of ‘bad’ pixels. For this, the signal of every pixel, S_*i,j*_, has been com­pared with the mean signal value across all pixels, 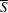 , and the number of unconnected or ‘dead’ pixels, 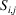 < 0.5 × 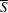 , together with the number of ‘hot’ and saturated pixels, 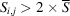 , have been evaluated. Any module showing any damage not identified during the visual inspection, *e.g.* unconnected pixels or a large cluster of ‘bad’ pixels, was rejected and not considered for the final detector assembly. An example of good quality and rejected modules is shown in Fig. 7 ▸.

### 3.2. Correction for bad pixels

**Figure 8:**
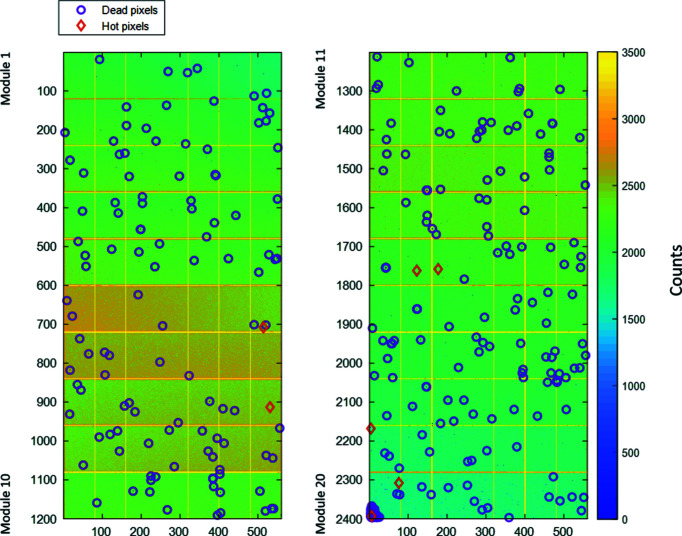
CirPAD image obtained from the scattering of a glass sample, with an energy of 18 keV and for an integration time of 60 s. The left column shows the first 10 modules and the right column shows the other 10 modules. The mean value of the signal per pixel is around 2000 counts. The bright vertical bars correspond to the ‘double’ pixel in the chip edge. The intense horizontal lines originate from the pixels close to the guard ring (Fig. 2 ▸). The edge of the last module (lower left corner) was damaged during installation. Note that the arrangement of the modules in the figure does not represent the physical detector geometry.

The final analysis and investigation of the CirPAD modules in terms of the number of bad pixels have been carried out for both types of beam calibration, SLOW and FAST, performed at 9 keV (see §2.1 for details). For this, a series of images has been acquired by illuminating the com­plete detector surface with 18 keV X-ray photons scattered from a glass sample placed in the direct beam path. 400 such ‘pseudo’-flat-field images have been recorded, with an exposure time of 60 s each, to reach at least an average 2000 counts by pixel. Due to the intensity gradient present in the beam over the entire CirPAD, the statistical analysis of defect pixels has been done chip-by-chip. For this, the series of 400 images has been averaged and the mean signal for each of 140 chips, 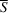 , has been calculated. All defect pixels have been identified and categorized by com­paring their value, 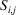 , following the same criteria as used in the laboratory qualification tests: the dead pixels when 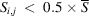 (including un­connected pixels) and the hot and saturated pixels when 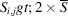 . The spatial distribution of these pixels across the detector surface is shown in Fig. 8 ▸. Furthermore, two additional criteria have been added in order to identify pixels that are within the acceptable range but are either undercounting (when 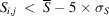 ) or overcounting (when 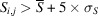 ), where 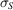 is the standard deviation. The undercounting and overcounting pixels are mostly due to an imperfect threshold calibration and can be com­pensated (§3.3) by the flat-field correction (Medjoubi & Dawiec, 2017 ▸). All identified malfunctioning pixels among all 20 CirPAD modules have been counted and are summarized in Table 3 ▸. The total number of unusable pixels (hot and dead) is very low, below 250 pixels in total, *i.e.* less than 0.02% of all pixels. Moreover, almost all pixels are calibrated correctly with less than 0.7% of pixels seeming to have a local threshold value set too high, which results in undercounting of the X-ray photons.

**Figure 9:**
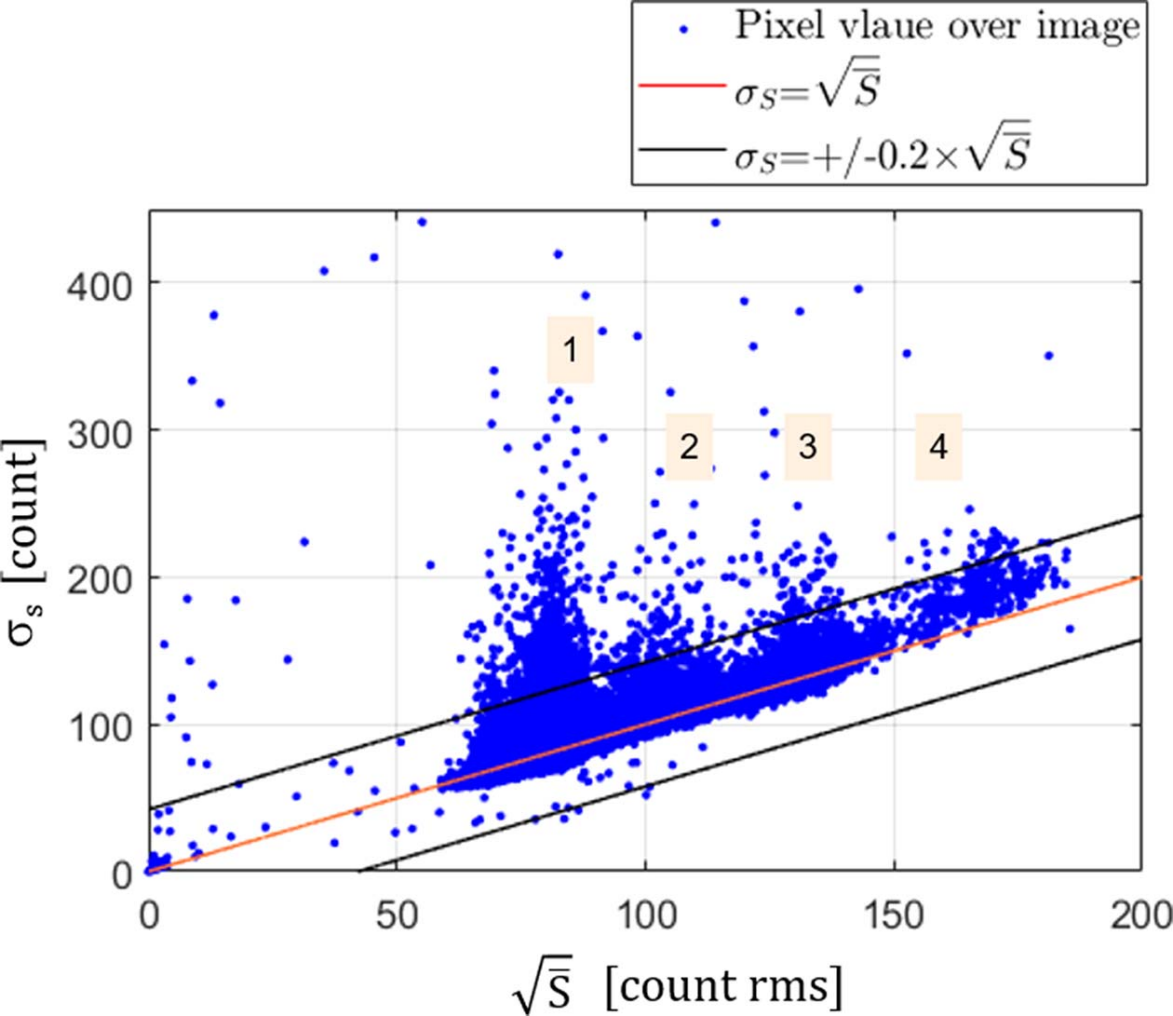
Standard deviation of all CirPAD pixels (averaged over 400 images) *versus* its mean intensity. The images were acquired with SLOW calibration and a counting time of 120 s, with an average of 6000 counts per pixel. Four groups of pixels are highlighted: (**1**) the largest number of pixels over the image; (**2**) the so-called double pixels; (**3**)/(**4**) pixels close to the guard ring. The Poisson distribution 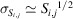 is shown as the red line and two black lines at the 20% limit that determines the acceptance levels.

Two similar series of 400 images have been acquired for SLOW and FAST calibrations. The distribution of the res­ponse of all pixels as the standard deviation, 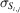 , as a function of the root square of the mean signal 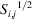 for every pixel has been plotted. Considering an ideal photon-counting detector, the fluctuation of the counts is limited by Poisson statistics, therefore, 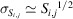 . The resulting distribution for SLOW calibration is shown in Fig. 9 ▸. One should note that the distribution has been calculated on raw data without any flat field and/or geometrical corrections and without suppressing (masking) bad pixels. The Poisson limited distribution is shown as the red line and a 20% margin region is highlighted with two black lines. As can be noticed, the majority of the pixels follow the Poisson limited statistics, with about 0.55% of pixels outside the defined range. The horizontal extension of the central cluster of values is due to a non-uniform illumination of the detector over the full 135° spanned angular range. Additionally, a smaller cluster of values with a larger mean signal is visible, due to the larger sensor pixels between adjacent readout chips (double pixels), as well as the guard ring around each sensor module. For the last two pixel families, counts coming from the larger sensor surface, thus with a higher mean signal, can be easily corrected by applying geometrical and flat-field corrections.

### 3.3. Homogeneity and flat-field correction

**Figure 10:**
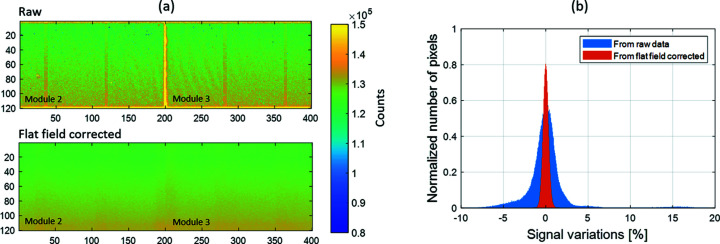
(*a*) Raw (top) and flat-field corrected (bottom) ROI images of 120 × 400 pixels between modules 2 and 3. (*b*) Histogram of the pixel-to-pixel variation for the com­plete detector for the raw and corrected images.

A flat-field correction of each raw image is essential to remove the fixed pattern noise due to sensor inhomogeneities and residual threshold dispersion increasing the range over which the detector performance is limited by Poisson statistics and the quality of the acquired data. For the best performance, images for the flat-field correction coefficients must be collected with significantly higher statistics and at the same energy as the images to which the correction is applied. In the presented example, the flat-field correction image has been obtained with a series of 100 images acquired at 10 keV energy and from the scattering of the air with the X-ray beam. The sum of all the images gives about 6.5 × 10^5^ photons s^−1^ pixel^−1^. The raw and flat-field corrected Region Of Inter­est (ROI) images of 120 × 400 pixels that cover the region between modules 2 and 3 are shown in Fig. 10 ▸(*a*). All the structures and artefacts on the raw images, such as the higher counting in the guard ring regions, correction for virtual pixels and sensor inhomogeneities, have been efficiently corrected and removed from the final image. Due to the size and geometry of the CirPAD, it is quasi-impossible to obtain a flat and uniform illumination of the whole detector surface. Therefore, a remaining gradient can be observed after flat-field correction. This gradient can also be corrected by plane or second-order polynomial levelling, in a similar manner to what is done in scanning probe microscopy. The resulting pixel-to-pixel signal variations have been significantly reduced from σ = 2.9% in the case of the raw data to σ = 0.36%. The overall homogeneity of the com­plete detector is very high, with a pixel-to-pixel signal variation within the ±1% range for all pixels, as shown in the histograms in Fig. 10 ▸(*b*).

### 3.4. High-count-rate performance

**Figure 11:**
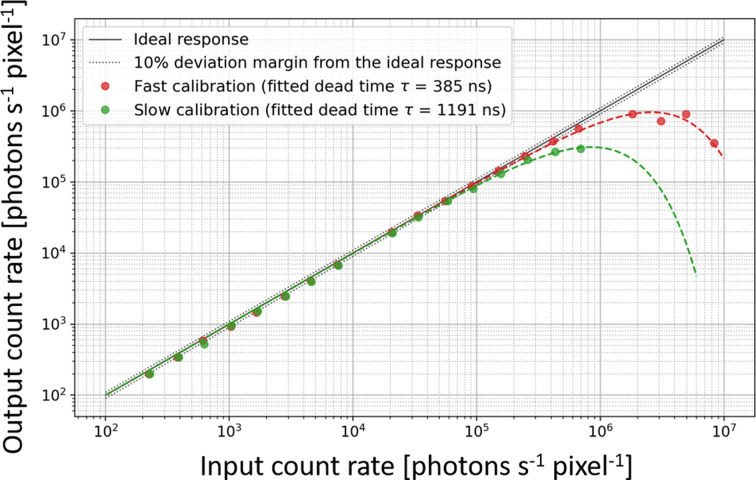
Count-rate linearity of the CirPAD measured for two different shaping times of the analogue front-end (FAST and SLOW).

The high-count-rate performance of the detector has been evaluated by illuminating the detector with a focused (∼250 µm × 180 µm FWHM, horizontal × vertical) mono­chro­matic beam of 18 keV energy and examining the response of a single pixel (the most intense one). For these measurements, the detector has been calibrated at 9 keV using the SLOW and the FAST methods that affects mainly the shaping time of the analogue pixel front-end. The X-ray beam has been gradually attenuated using a set of Cu foil absorbers and the real number of photons has been calculated with a calibrated photodiode placed close to the CirPAD. The measured count rates of the detector for two calibrations are shown in Fig. 11 ▸.

The two experimental curves have been fitted with the paralyzable detector model (Knoll, 2000 ▸) defined as:

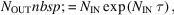

where *N*_OUT_ is the output count rate, *N*_IN_ is the input photon rate and τ is the detector dead time. For the shaping times of the two detectors, the measured dead times are 385 and 1191 ns for FAST and SLOW calibrations, respectively. This corresponds to maximum count rates of 2.5 × 10^6^ and 8.4 × 10^5^ photons s^−1^ pixel^−1^, respectively. The linear region, de­fined as a 10% deviation from the ideal response, is estimated at 2 × 10^5^ and 8 × 10^4^ photons s^−1^ pixel^−1^ for the FAST and SLOW settings, respectively.

### 3.5. CirPAD short-gate performance for time-resolved experiments

**Figure 12:**
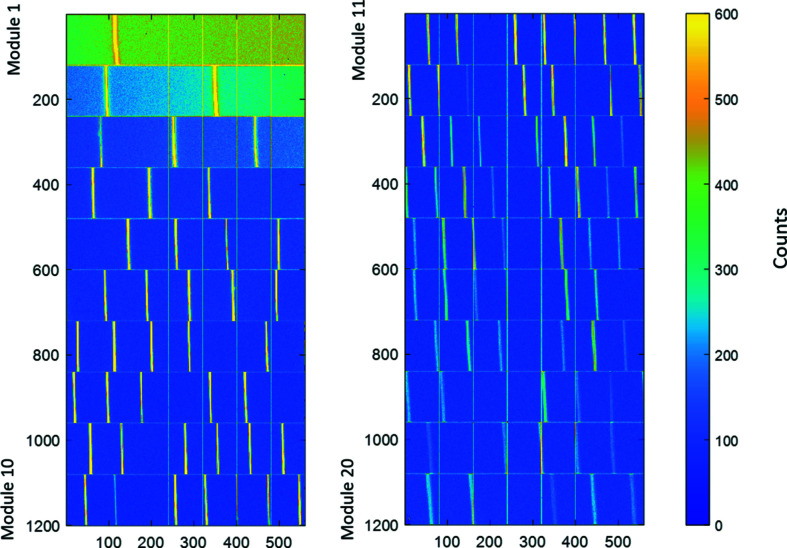
Image of the LaB_6_ powder recorded from the CirPAD using X-ray photons of 17.94 keV (10 s acquisition time). The modules index (from 1 to 20) increases from top to bottom for each panel.

The short-gate performance of the detector over all modules has been evaluated to ensure the capability of the CirPAD to record short intensity variations over all modules. For this, diffraction diagrams similar to those shown in Figs. 12 ▸ and 13 ▸ have been collected from the CirPAD while the storage ring operated in so-called hybrid-filling mode. At Synchrotron SOLEIL, the hybrid-filling mode consists of an isolated electron bunch (of 60.8 ps length) in the middle of one empty quarter of the storage ring and the three remaining quarters are filled with almost uniformly distributed multi-bunches. The time gaps between the isolated bunch and multi-bunch sections are 147.64 ns. This configuration is shown schematically in Fig. 14 ▸(*a*). The smallest gate opening for the detector (100 ns, see Table 1 ▸) was used for the acquisition of this data set. Because the number of photons detected per integrated diffraction ring in a single 100 ns length gate opening is low (due to pulse-shaping dead time; see §3.4), a series of about 1.5 × 10^6^ images at the same Δ*t* delay (synchronized with the electron bunches) have been accumulated into a single image. The gate signal has been generated by the TimBeL synchronization card (Ricaud *et al.*, 2011 ▸) and its Δ*t* delay has been increased progressively in steps of 10 ns to cover a delay range of more than one com­plete storage ring revolution period, which is about 1.18 µs [Fig. 14 ▸(*b*)].

For each resulting CirPAD image and Δ*t* delay, the corresponding diffractograms are extracted following the procedure detailed in §4. The integrated intensity of each diffraction peak is extracted and its evolution *versus* the applied delay allows the time structure of the electron current in the storage ring to be described. As an example, the resulting XRD signal *versus* the applied delay between two different peaks, measured on two different modules of the CirPAD, is shown in Fig. 14 ▸(*c*). The measurement is the result of the convolution of the ring current profile [Fig. 14 ▸(*a*)] with the gate function. The single bunch can be easily determined and the presence of an almost constant number of counts (small plateau) in that region confirms the good separation of the isolated bunch. The presence of the three quarters in the ring-filling pattern can be detected as well [*cf*. intensity dips at delays Δ*t* ≃ 0, 300 and 600 ns; Fig. 14 ▸(*c*)], due to the presence of filling-pattern non-uniformities at these locations [Fig. 14 ▸(*b*)]. Moreover, a careful examination of the signals originating from the approxi­mately 100 measured diffraction peaks shows that the module-to-module variation of the temporal response is in the order of the minimum delay step used for the measurement.

## 4. First X-ray diffraction image

As mentioned above, the CirPAD consists of a matrix of 120 × 560 × 20 = 120 × 11200 pixels. This data set is saved as a matrix of (2400 × 560) values, corresponding to the 20 XPAD-S70 modules (each of size 120 × 560). To extract the measured scattered intensity *versus* the scattering angle (denoted by 2θ from here on), several points are to be considered:

(i) The precise positioning of the pixels on the chips has to be known. This particularly includes double pixels, damaged or too close to the guard ring, since they can be masked and will not contribute to the reconstructed diffractograms.

(ii) The intensity response of each pixel, com­pared to its neighbours, has to be known (nonlinearity). This can be accessed by illuminating the detector with a broad-size and uniform-intensity X-ray beam, and recording images with good statistics (flat-field correction). The intensity of each pixel can then be renormalized during a sample measurement.

(iii) The orientation of the detector in space (lateral and angular positions, for each of its 20 modules) with respect to the direction of the incident X-ray beam and with respect to the sample position has to be known as accurately as possible. By geometrical transformations, Cartesian coordinates can then be calculated for each pixel. The intensity of each pixel can then be related to its angular position in space (or its polar coordinates, scattering angle 2θ and elevation angle ψ) and the corresponding 2D regrouping image (sometimes called ‘caking’) can be generated (details are given in Appen­dix *A* [App appa]). A subsequent mathematical procedure allows the regrouping of pixels belonging to a diffraction ring in order to retrieve diffractograms. These issues are described in detail in Kieffer *et al.* (2012 ▸).

The last point is difficult to deal with using only the values obtained from metrology (mechanical mounting). To overcome this issue, an X-ray diffraction pattern from a NIST- certified powder, namely Lanthanum Hexaboride LaB_6_ (Black *et al.*, 2020 ▸), was used. The powder was sealed in a ∼250 µm inter­nal diameter capillary which was rotated continuously at a frequency of about 2 Hz during the data collection to limit an effect potentially originating from the orrientation of the crystallites (texture). The typical integration time per image was in the 1–10 s range and the obtained recorded image can be seen in Fig. 12 ▸. The energy used for the measurement was 17.94 keV (or wavelength 0.06911 nm) and the beam size was about 250 µm × 200 µm (FWHM, horizontal × vertical). CirPAD was beam calibrated using calibration at half of the working energy (9 keV) and using the SLOW settings.

**Figure 13:**
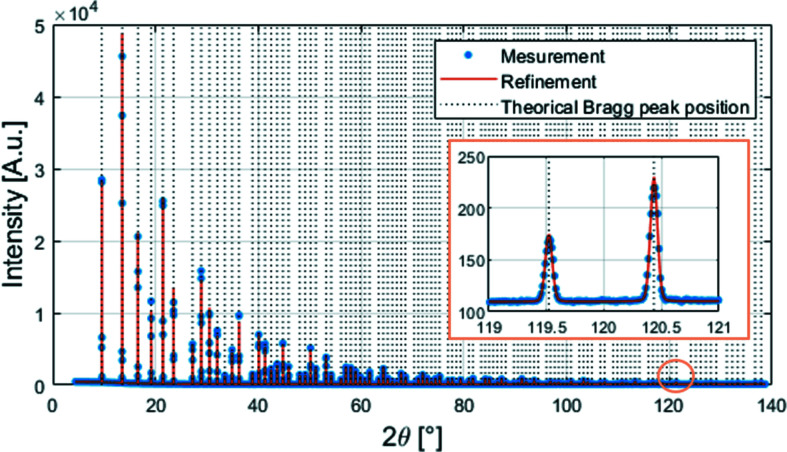
Powder diffraction pattern from a 0.25 mm diameter capillary filled with LaB_6_ reference powder from NIST measured at 17.94 keV (blue points). Two images were recorded at two detector positions for a total acquisition time of 2 × 10 s and the selected threshold calibration of the detector was with beam mode and SLOW settings. The red line is the whole pattern structural refinement performed using the NIST reference values. The inset shows a magnification of two peaks at high angles.

**Figure 14:**
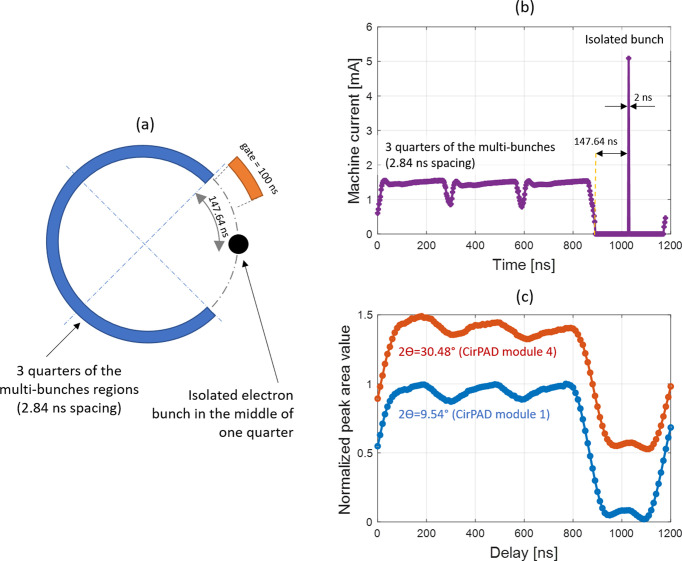
(*a*) Schematic view of the bunch distribution in the hybrid-filling mode at Synchrotron SOLEIL. (*b*) The corresponding machine current distribution. (*c*) Example of the intensity reconstructed for two diffraction peaks (2θ = 9.54 and 30.48°, thus two different CirPAD modules). Each point corresponds to a different delay value separated by a 10 ns step. The intensity corresponds to the integrated signal (area) over the corresponding XRD peak from the LaB_6_ powder. Data sets are scaled and shifted vertically for better visibility.

As described above (point iii), the X-ray diffraction pattern is obtained from at least two images similar to the one above (Fig. 12 ▸) to eliminate dead zones. In order to obtain a diffractogram, the geometry of each module used as a starting point is that from metrology. By slightly adjusting the position and orientation of each of the 20 modules, the positions of the XRD peaks are matched to their theoretical values. This procedure allows the orientation of the detector to be finely tuned and the exact geometry which will be used for subsequent measurements to be obtained. The com­parison of the reconstructed diffractogram and the theoretical positions of the XRD peaks is shown in Fig. 13 ▸. The quality of the data is evaluated using the whole powder pattern matching method (Le Bail *et al.*, 1988 ▸) and structural refinements (Rietveld, 1969 ▸), performed using the powder diffraction software *FullProf* (Rodríguez-Carvajal, 1993 ▸; Roisnel *et al.*, 2000 ▸). The background adjustment was made with a series of selected points and, after indexing the pattern, the peak shapes were fitted by a specific profile function (Npr = 7; Thompson–Cox–Hastings pseudo-Voigt convoluted with axial divergence asymmetry function) (Finger *et al.*, 1994 ▸). The structure was satisfactorily refined, as shown by the values of the parameters evaluating the quality of the fit (Table 4 ▸). The difference between the experimental data and the calculated pattern reveals only a few deviations in the peak position at high angles, but there are no differences between the observed and the calculated intensities.

**Figure 15:**
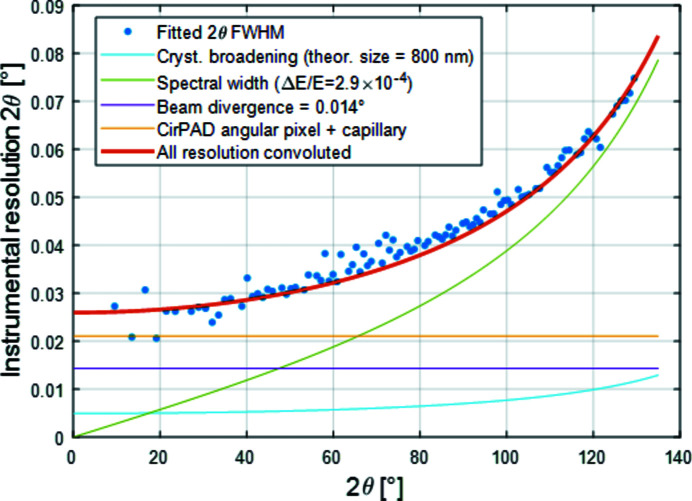
FWHM of the measured diffraction peaks (blue dots) deduced from the data set shown in Fig. 13 ▸. The different contributions to the peak broadening are highlighted: incident photon spectral width (∼3 × 10^−4^, green), incident beam divergence (0.014°, magenta), crystallite size broadening (cyan), point spread function of the XPAD and the beam/capillary size (orange). The modelled resolution function (red curve) is calculated as a convolution of the various contributions.

The diffractograms obtained for the LaB_6_ reference also allow the extraction of the instrument resolution function from a single data set (Guinebretière *et al.*, 2005 ▸). This is useful information when per­for­ming XRD measurements on polycrystalline samples, since it allows the data to be corrected prior to ex­trac­ting, for example, reliable values for crystallite size, micro­strain, *etc*. Fig. 15 ▸ reports the width of the XRD peaks (FWHM values, Gaussian fit) as a function of their respective 2θ position. The observed dependency can be modelled using the various contributions present (incident beam divergence, detector point spread function – pixel angular opening, mono­chromaticity/spectral width, sample/capillary size/…, *etc*.).

## 5. Summary and perspectives

The CirPAD developed, fabricated and installed on the DiffAbs beamline is a new large 1.4 M hybrid pixel detector consisting of the assembly of 20 XPAD-S70 modules along a circular arc, with a 645 mm radius. This particular shape makes this 2D detector unique thanks to its ability to collect X-ray diffraction images in a 135° angular range, as well as the overlapping between two modules for reducing areas without any signal counting (dead zones). All supports, frame, arch and crane allow a good mechanical stiffness to be provided, with a deformation (in all directions) less than the size of a single pixel.

In order to determine the performance and quality of images obtained from the CirPAD, the uniform response has been verified. The threshold calibration with beam FAST and SLOW settings has been performed to qu­antify the number of bad pixels. A low number of defective pixels has been measured: 0.09% of dead pixels for the FAST calibration mode and close to 0.02% for the SLOW one, and only a few hot pixels (0.0001%) regardless of the mode. Flat-field ima­ging and mapping of all the defective or noisy pixels have been performed, and both will be used to correct the data sets. The limit of the count rate has been measured and reaches 2.5 × 10^6^ and 8.4 × 10^5^ photons s^−1^ pixel^−1^ for FAST and SLOW calibration, respectively.

The diffraction images obtained from the CirPAD using the LaB_6_ reference powder allowed the architecture of the detector, including the electronics, the geometry of the 20 modules and their synchronized readout to be verified. From this diffraction image, the metrology of each module has also been realized such that the obtained intensity *versus* scattering angle diffractogram is in very good agreement with the expected reference data set. This reconstruction process of the diffraction pattern, from geometric corrections and calibration to diffractograms, has been validated by a correct Rietveld refinement of the LaB_6_ reference powder. This approval promises good refinement on various samples.

One of the major properties of the CirPAD is based on the capability to drastically reduce the acquisition time. In standard operation mode, the exposure can be reduced to a few ms (*cf*. Table 1 ▸), while the synchronous acquisition mode allows exposures (gates) as short as 100 ns, in a stroboscopically image accumulation mode (Single Bunch Mode). The feasibility of such pump–probe experiments was illustrated by using the LaB_6_ calibration powder and detecting the isolated electron bunch in a particular filling pattern of the synchrotron ring, all while different diffraction peaks were followed simultaneously over a wide domain of the scattering angles. This makes this detector adapted to a number of synchronized time-resolved pump–probe X-ray diffraction experiments, with the major advantage of giving access to extended *q*-range diffractograms.

The use of the CirPAD and its great inter­est for materials science and physical chemistry scientific applications, including highlights/scientific cases showing the capabilities of the CirPAD for the structural characterization of polycrystalline, textured or amorphous materials, will be the subject of a second article.

## Supplementary Material

Movie for Appendix A. Data representation in spherical coordinates of the XRD rings for the reference LaB6 powder performed wiith CirPAD. DOI: 10.1107/S1600577521012492/gy5028sup1.avi

## Figures and Tables

**Figure 16:**
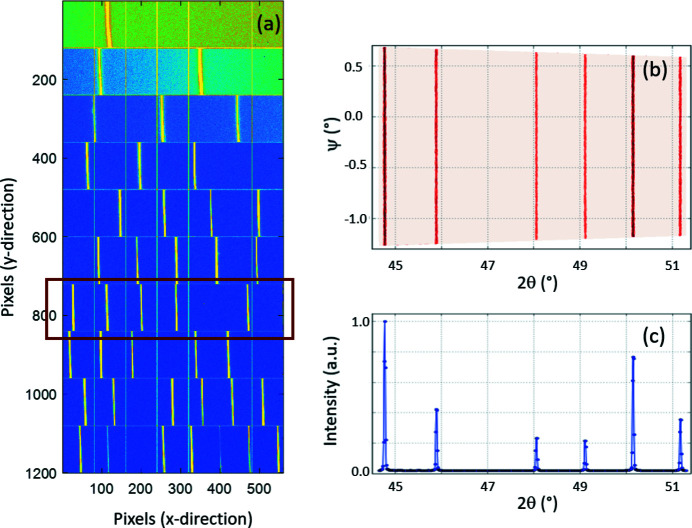
(*a*) Image of the data acquired with the CirPAD (*R_z_* = 0°), represented similar to Fig. 13 ▸. Only the first 10 modules are shown, with module 1 being located at the top of the image; (*b*) 2D regrouped image (intensity *versus* scattering angle 2θ/azimuthal angle ψ) for the particular module (number 7) highlighted in part (*a*). In these coordinates, diffraction rings appear as straight vertical lines (2θ = constant). The intensity colour scale is logarithmic. (*c*) The corresponding reconstructed 1D powder diffraction pattern (logarithmic intensity scale).

**Figure 17:**
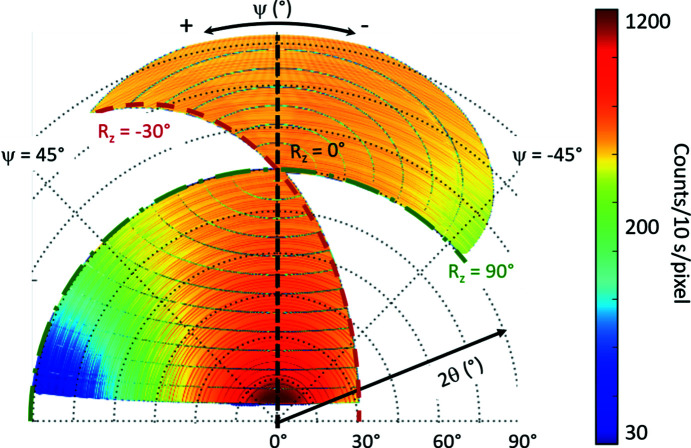
Polar representation (radius is 2θ and azimuthal is ψ) of the accessed angular volume during a measurement with the CirPAD for *R_z_* = −30 to +90° (step of 1°). The trajectories, in angular space, of the reconstructed data from single CirPAD images, are shown for the cases *R_z_* = −30, 0 and 90° (red, black and green dashed curves, respectively). The intensity colour scale (from blue to red) is logarithmic. The presence of the missing data corresponding to inter-module gaps appears as curved grey regions. Due to the very large size, the data were relatively strongly binned (∼20 × 20 pixels, *i.e.* ∼0.2 × 0.2° angular opening) for ease of being shown.

**Table 1:** Characteristics of the single XPAD-S70 detector module based on the XPAD3.2 readout chip

Sensor type	1 module with 7 readout chips (XPAD3.2)
	Silicon (500 µm thick)
Pixel size	130 µm × 130 µm
Efficiency	99.9% at 8 keV
	42% at 18 keV
Total active surface	80 × 120 × 7 = 67200 pixels
	15.60 mm × 75.14 mm
Energy range	7–35 keV
Threshold adjustment	4–30 keV
Threshold dispersion	150 eV
Maximum count rate	2.5 × 10^6^ photons s^−1^ pixel^−1^ (FAST)
(see §3.4)	8.4 × 10^5^ photons s^−1^ pixel^−1^ (SLOW)
Linearity limit	2 × 10^5^ photons s^−1^ pixel^−1^ (FAST)
(see §3.4)	8 × 10^4^ photons s^−1^ pixel^−1^ (SLOW)
Dynamic range	32 bits (in Standard Mode with OVF)
Frame rate	250 Hz continuous
	700 Hz by bunches of 980 images
Minimum gate width	100 ns (see §3.5)
Spatial resolution	1 pixel (Point Spread Function)

**Table 2:** Main characteristics of the CirPAD

Modules	20 XPAD-S70
	20 × 120 × 560 pixels
	20 × 15.6 mm × 75.14 mm
Total pixels	1344000 pixels
Module dead pixel	7 pixels
Total dead zone (without edge)	19 pixels
Detector radius	645 mm
Experimental angular range (meridian × equator)	135° (2θ) × 1.38°
Total circumference	1519 mm
Angular resolution (regular/‘double’ pixel)	0.0115°/0.0287°
Module angle (*versus* neighbour)	6.74°
Angular acceptance of one module	6.67° × 1.38°

**Table 3:** Total number of CirPAD defects and the less good calibrated pixels counted for SLOW and FAST calibration

Category	Criteria	Number of pixels with SLOW beam calibration	Number of pixels with FAST beam calibration
Dead	S_{i,j} \ \lt \ 0.5 \times {\overline S}	240 (0.02%)	1280 (0.09%)
Undercounting	S_{i,j} \ \lt \ {\overline S} - 5 \times \sigma _{S}	9244 (0.69%)	48762 (3.63%)
Overcounting	S_{i,j} \ \gt \ {\overline S} + 5 \times \sigma _{S}	263 (0.02%)	866 (0.06%)
Hot	S_{i,j} \ \gt \ 2 \times {\overline S}	3 (<0.0001%)	10 (0.0001%)

**Table 4:** Parameters used for the structure refined calculation

Wavelength (Å)	Flat-field correction	χ^2^	Unweighted *R*_p_	Weighted profile *R*_wp_	Expected *R*_exp _	Bragg *R* factor	RF factor
0.691105	Yes	6.44	21.8	29.0	11.44	4.591	1.696

## Appendix A Example of the workflow used for 2D reconstruction from CirPAD data

Figs. 16 ▸ and 17 ▸ show examples of the workflow used to re­con­struct data acquired with the CirPAD (case of the LaB_6_ NIST calibration powder). The pixel coordinates of each module for each image [Fig. 16 ▸(*a*)] are transformed into angular coordinates and the corresponding 2D regrouped image (sometimes called ‘caking’) can be generated [Fig. 16 ▸(*b*)]. In this representation (2θ = scattering angle and ψ = azimuth on the XRD ring), the XRD rings appear as vertical lines (2θ = constant). The data can be converted into 1D diffractograms (intensity *versus* scattering angle 2θ) [Fig. 16 ▸(*c*)] by performing the data regrouping (see also Fig. 13 ▸).

The wide range (∼120°) available for rotation around the *z* axis (*R_z_*, see label **6** in Fig. 6 ▸), combined with the about 135° × 1.38° angular acceptance of the detector (see Table 2 ▸), allows for 2D mapping of large angular regions. An example of a measurement performed in the range *R_z_* = −30° to +90° (with a step of 1°) is shown Fig. 17 ▸. The resulting individual 2D regrouped images are combined into angular maps (intensity *versus* 2θ *versus y*, about 1.6 × 10^8^ pixel points), which access more than 1/8 of the sphere in angular coordinates (2θ ∼ 0–135° and ψ ∼ 0–90° at least). The volume accessed *via* this approach is illustrated in Fig. 17 ▸, together with the reconstructed sections corresponding to particular *R_z_* positions of the CirPAD and the XRD rings for the reference LaB_6_ powder. Data representation in spherical coordinates can be found in the supporting information.
